# Dual-task meta-auxiliary learning in laparoscopic cholecystectomy

**DOI:** 10.1007/s11548-025-03442-w

**Published:** 2025-06-26

**Authors:** Rui Guo, Conor Perreault, Benjamin Mueller, Xi Liu, Anthony Jarc

**Affiliations:** Digital Solutions, Intuitive Surgical, Peachtree Corners, GA 30092 USA

**Keywords:** Machine learning, Anatomy segmentation, Clinical milestone, Meta-auxiliary learning, Vision transformer

## Abstract

****Purpose**:**

Artificial intelligence is transforming surgical practices by improving procedural quality and decision-making. Machine learning-based video analysis can reliably identify surgical milestones, enhancing contextual understanding for surgeons. This study proposes a novel framework for detecting critical view of safety (CVS) in robot-assisted laparoscopic cholecystectomy (RLC) to improve procedural safety.

****Methods**:**

We present a meta-auxiliary learning framework that delicately combines milestone recognition and anatomical segmentation to enhance contextual awareness. The framework addresses label imbalance by facilitating knowledge sharing across tasks, ensuring balanced optimization. A curated RLC dataset was utilized to evaluate CVS identification and multi-instance segmentation performance.

****Results**:**

The proposed method achieved an F1 score of 78% for CVS detection and a mean IOU of 83.9% for anatomical segmentation, demonstrating its efficacy in complex surgical environments.

****Conclusion**:**

This framework establishes a new paradigm for surgical video analysis by integrating milestone detection and segmentation. Its ability to enhance decision support and procedural review in RLC highlights its potential for broader adoption in clinical practice.

## Introduction

Laparoscopic cholecystectomy (LC), or the surgical removal of the gallbladder, has been widely used as a minimally invasive surgical technique in the past few decades [1–3]. With the advancement of surgical robots and computer-assisted technologies, robot-assisted LC has emerged as a promising approach for improving surgical precision and patient outcomes. One of the key challenges in robot-assisted LC is the recognition of clinical milestones, such as the Critical View of Safety (CVS) that is essential for minimizing the risk of bile duct injuries and ensuring the safety of the procedure. In clinical settings, CVS achievement is typically measured by performing surgeons who visually inspect the surgical field prior to proceeding with the surgery. The CVS achievement standard is widely accepted, and defined by SAGES [4] as three criteria:

- **C1: The hepatocystic triangle is cleared of fat and fibrous tissue.** The hepatocystic triangle is defined as the triangle formed by the cystic duct, the common hepatic duct, and inferior edge of the liver. The common bile duct and common hepatic duct do not have to be exposed.
- **C2: The lower one third of the gallbladder is separated from the liver to expose the cystic plate.** The cystic plate is also known as liver bed of the gallbladder and lies in the gallbladder fossa.
- **C3: Two and only two structures should be seen entering the gallbladder.**

**Fig. 1:**
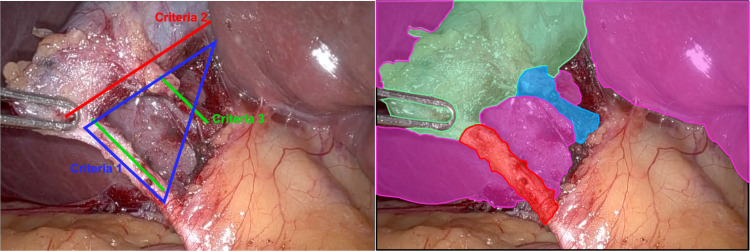
An anatomical view in Cholecystectomy. Left: The criteria of Critical View of Safety (CVS); Right: Masks of major anatomies. Liver (magenta), Gallbladder (mint), Cystic Duct (red), Cystic Artery (light blue)

Although the criteria are well defined, CVS recognition in the clinical process is still imperfect due to subjective judgment, camera misalignment, and inter-observer variability. These factors can impact the accuracy, quality, and consistency of the surgery [5] (Fig. 1).

The CVS criteria clearly demonstrate that CVS achievement is highly correlated with the spatial status of the major anatomies in Cholecystectomy. This highlights another task: identifying anatomical structures of interest, such as the liver, gallbladder, cystic duct, and cystic artery, which can potentially improve milestone recognition. Typically, surgeons try to achieve a clear view of these key anatomies to avoid any confusion in their decision-making. Spatial segmentation in this scenario contains crucial context for understanding both the complexity of the case and the progress of key milestones, and therefore can help achieve a successful surgery and avoid potential complications. However, manual segmentation is time-consuming and prone to human error, while automatic segmentation algorithms often struggle with the variability and complexity of the anatomical structures. In the general clinical algorithm development, multi-task labels are often collected unevenly. The imbalanced label for the dataset discourages learning in a simultaneous way. For our case, the spatial mask for major anatomies in Cholecystectomy is much more expensive than the CVS status to annotate, making it currently impossible to approach this as a dual-task problem.

In this paper, we propose a novel approach for addressing both challenges in LC, by combining anatomy segmentation and clinical milestone recognition using meta-auxiliary learning strategy. Meta-auxiliary learning is a form of meta-learning that involves training a meta-model to predict the performance of auxiliary tasks, which are related but different from the main task of interest while leveraging the correlated knowledge discovered from auxiliary task to enforce the capability of the main task. In our case, the main task is clinical milestone recognition, while the auxiliary task is anatomy segmentation.

Our approach consists of two stages. In the first stage, we train a deep neural network to perform anatomy segmentation using a large dataset of annotated laparoscopic videos. We evaluate the segmentation performance on a separate test set, and compare it with other state-of-the-art segmentation methods. In the second stage, we use the segmentation as an auxiliary task in training a meta-model for clinical milestone recognition learning. The meta-model takes the laparoscopic videos as input and predicts the probability that each clinical milestone is completed. We evaluate the milestone recognition performance on a separate test set, and compare it with other state-of-the-art methods.

Our results demonstrate that our proposed architecture achieves superior performance in both anatomy segmentation and clinical milestone recognition, compared to other state-of-the-art methods. We also conduct ablation studies to investigate the contribution of each component in our approach. Our approach has the potential to improve the quality and efficiency of robot-assisted LC, and can be extended to other robot-assisted surgical procedures in hierarchical tasks.

## Related works

Recent advancements in computer-assisted interventions for cholecystectomy have focused on automating the detection and evaluation of the Critical View of Safety (CVS). Initial efforts utilized binary image classification models to assess CVS achievement and formalize criteria for automatic CVS grading. Tokuyasu et al. [6] introduced a landmark detection system that focused on identifying anatomical features such as the common bile duct and cystic duct. Expanding on this foundation, Mascagni et al.[7] combined hepatocystic anatomy segmentation with CVS classification, bridging the gap between image segmentation and CVS detection.

Building further, Madani et al. [8] proposed an automated system for early identification of safe and unsafe dissection zones during cholecystectomy. Owen et al. [3] pursued a similar objective by emphasizing anatomy segmentation around CVS, with a particular focus on identifying the cystic artery and cystic duct. This work diverged in its methodology by adopting an alternative framework for image segmentation and milestone recognition.

Beyond these domain-specific approaches, broader advancements in machine learning have significantly influenced clinical research. Transformer-based architectures such as TransUNet [9] have demonstrated exceptional capabilities in extracting attention-focused features for medical image segmentation. Similarly, TimeSFormer [10] has gained attention for disentangling temporal correlations in sequential video data, proving effective in action classification tasks. Meanwhile, meta-learning and auxiliary learning frameworks [11–13] have shown promise in multi-task learning by leveraging shared representations across tasks to enhance individual task performance.

To the best of our knowledge, this work represents the first attempt to simultaneously model CVS recognition and primary anatomy segmentation within a meta-auxiliary learning framework. By integrating the latest advancements in AI, our approach addresses the limitations of previous methods, offering a robust solution for surgical video analysis.

## Methodology

We model the CVS detection as a multi-label classification problem given sequential video frames as the input. The encoded representation from the input also supports an auxiliary task that segments the major anatomies. Most existing solutions using multi-task scheme that build the two-branch architecture directly learn the mapping function in an inefficient way. In this section, we present an approach by constructing a meta-auxiliary learning that solves the data bottleneck in clinical research. There are several main benefits and novelties: first, a novel application of the TimeSFormer architecture as the backbone encoder, an architecture that has proven performance in disentangling the temporal information in video sequences. Second, the plain dual-task method is replaced by meta-auxiliary learning, focusing on the primary CVS detection task with improved efficiency by leveraging the auxiliary segmentation task to explore the spatial correlation between the tasks. Third, the introduced meta-auxiliary learning scheme also helps in the treatment of imbalanced data labeling via joint meta-learning, which limits many machine learning adaptations in video assisted clinical research.

**Fig. 2:**
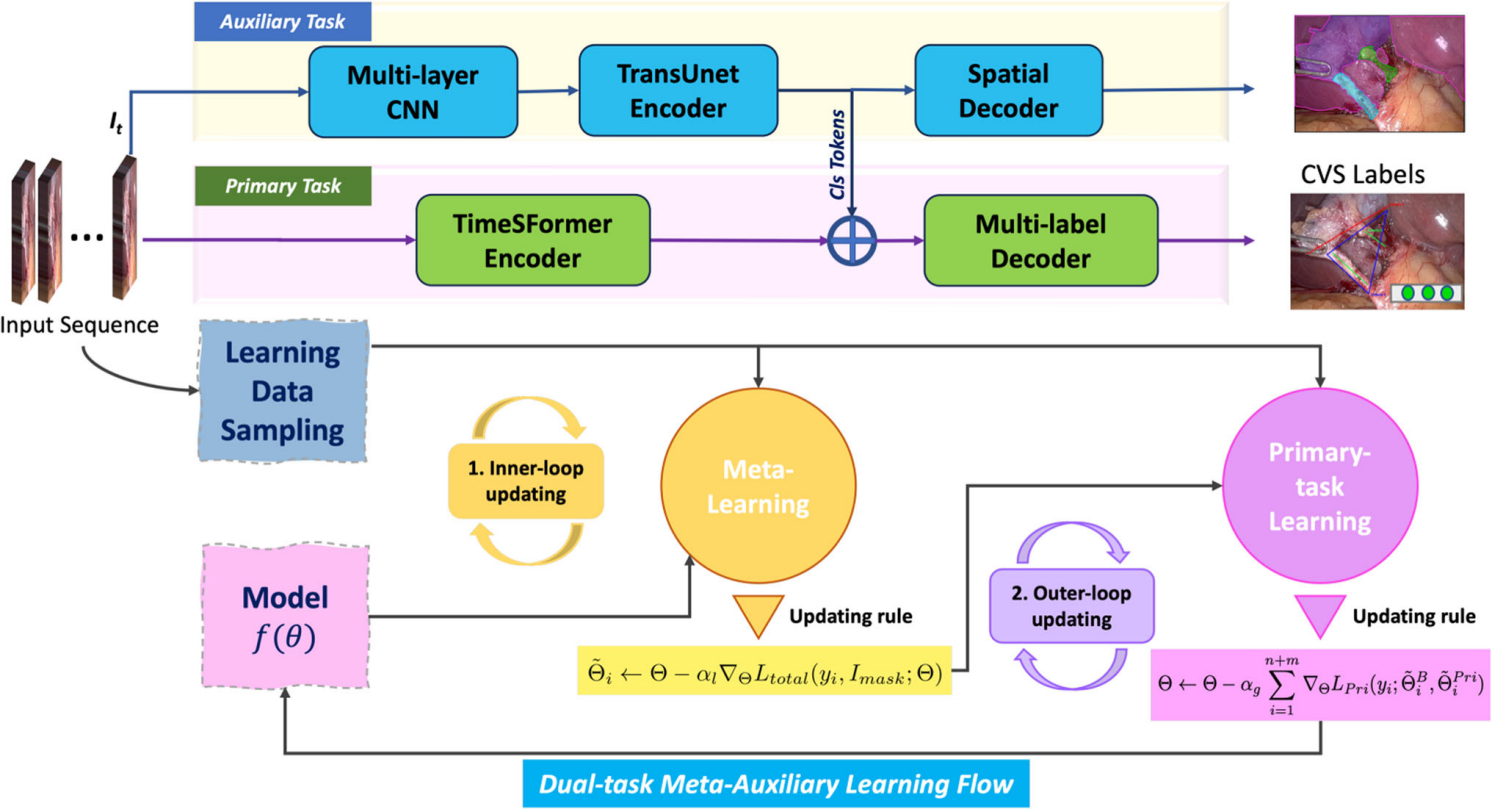
Illustration of two-branch Meta-auxiliary learning (on the top) and dual-task learning flow (at the bottom). In the learning process, the model firstly obtain the adaptive parameters based on the total loss in the inner-loop updating with dual-labeled sampled data. Then, model weights are refined at the outer-loop based on primary loss targeting to acquire high performance on the primary task with entire sampled data

### Model architecture

In the following, we present the proposed two-branch architecture in details in Fig. 2. It consists of a primary branch for the CVS detection task and an auxiliary branch for the supervised anatomy segmentation task.

**Backbone TimeSFormer network:** The backbone network is a transformer-based model, designed to process a Cholecystectomy procedure video sequence efficiently to produce the encoded representations. This network adapts the standard TimeSFormer [10] architecture to video by enabling spatiotemporal feature learning directly from a sequence of frame-level patches. The network stacks convolution, MLP layers, and more importantly, space-time self-attention layers all together to enforce the exploring of recurrent patterns in video representation learning. The multi-scale and multi-head tokens are processed through MLP into an integrated feature vector.

**Primary CVS detection task:** Following the core network, a final embedding layer is obtained to formulate the classification token. On top of the TimeSFormer output, a one-hidden-layer MLP is appended to convert the representation vector into the token and ready to be fed into the classification head. For CVS task, we create 3 classes corresponding to criteria 1, 2 and 3, respectively. In order to enhance the network’s ability to capture intermediate features in the spatial dimension, the primary branch feature vector is concatenated with the class tokens from the auxiliary branch before decoding.

**Supervised anatomy segmentation in the auxiliary task: ** An appropriately chosen auxiliary task could significantly enhance the primary task by imparting additional insights into the input data. In clinical scenario, the concept of CVS in Cholecystectomy contains a strong correlation to the spatial layout of the major anatomies, such as liver, gallbladder, cystic artery and cystic duct. The auxiliary task is designed to capture intrinsic attributes of these major anatomies in spatial domain, thereby aiding the primary task.

The segmentation branch is inspired by TransUnet [9, 14], which is popularly adopted for medical image segmentation. For the input image stack, the very last frame $i_t$ is processed through convolutional layers and a transformer unit. The comprehensive feature representation is further processed with a reversed upsampling cascade. At different scale levels, hidden features generated from the encoding CNN are concatenated with the upsampled feature tensor through a skip-connection. The cascaded upsampling blocks are instantiated by stacking a $2\times$ upsampling operator, a $3\times 3$ convolution layer, and a ReLU layer successively. The feature representation is then recovered back to the original resolution of the input image.

**Dual-task joint learning:** In the learning process, we define the network in parametric way as $\Theta = \left( \Theta ^B, \Theta ^{Pri}, \Theta ^{Aux} \right)$, where $\Theta ^B$ denotes the shared weights, $\Theta ^{Pri}$ and $\Theta ^{Aux}$ are the task-specific weights for the primary CVS detection branch and the auxiliary segmentation branch, respectively. For primary CVS detection task, we adopt the binary cross-entropy loss (BCEwithLogits) that is defined on the multi-class problem and denote it as $L_{Pri}$. Similarly, for the segmentation auxiliary task, we take focal loss and denote it as $L_{Aux}$. The joint learning will aim to optimize the total loss over the dual tasks,

1 $$\begin{aligned} \begin{aligned}&L_{total}\left( y, I_{mask}; \Theta ^B, \Theta ^{Pri}, \Theta ^{Aux} \right) \\&\quad = L_{Pri}\left( y; \Theta ^B, \Theta ^{Pri} \right) \\&\quad \quad +L_{Aux}\left( I_{mask}; \Theta ^B, \Theta ^{Aux}, \Theta ^{Pri} \right) \end{aligned} \end{aligned}$$

where *y* represents the multi-class CVS label, and $I_{mask}$ denotes the groundtruth segmentation mask for the defined anatomies. In practice, the total loss is weighted by 0.9 and 0.1 on $L_{Pri}$ and $L_{Aux}$ to balance on their scales.

### Dual-task meta-auxiliary learning

The model derived from joint training exhibits suboptimal performance, primarily due to its exclusive reliance on well-scaled data with balanced labels [11, 15]. In response, we introduce meta-auxiliary learning to guide the acquisition of the learning momenta that harmonize between tasks and imbalanced labels. This often involves refining and customizing a learning scheduler [13], which is a non-trivial task that we would like to avoid. A more dedicated way is a bi-level optimization strategy, in which the inner-loop learning is focus on the meta parameters for the entire system, and leveraging it to boost the primary task on later outer-loop updating.

**Meta-auxiliary training:** We propose to integrate meta-auxiliary learning with a dedicated data sampling strategy. Given the imbalanced dataset, we stack **m** data with paired labels (CVS label and segmentation mask label in our case) and batch them with **n** data with only the primary task label (CVS label). The learning scheme is a bi-level optimization, as shown in Fig. 2. For an inner-loop process, $L_{total}$ is used to train on the *m* dual-labeled data points, to acquire a general spatial-temporal knowledge from both tasks. For each sampled image frame *i* in a batch, we have

2 $$\begin{aligned} \tilde{\Theta }_i \leftarrow \Theta - \alpha _l \nabla _\Theta L_{total}(y_{i}, I_{mask}; \Theta ) \end{aligned}$$

where $\alpha _l$ is the meta-learning rate. $\tilde{\Theta }_i = (\tilde{\Theta }_i^{B}, \tilde{\Theta }_i^{Pri}, \tilde{\Theta }_i^{Aux})$ is the adaptive neural network parameters. For the outer-loop process, the entire batched data $m+n$ is in effect and the primary objective is then achieved by performing gradient descent on $L_{Pri}$. It steers the learning toward an optimal point for the primary task.

3 $$\begin{aligned} \Theta \leftarrow \Theta - \alpha _g \sum _{i=1}^{m+n} \nabla _\Theta L_{Pri}(y_{i}; \tilde{\Theta }_i^B, \tilde{\Theta }_i^{Pri}) \end{aligned}$$

where $\alpha _g$ is the primary learning rate. In practice, when we use batchsize = 16, and m:n = 5:3, the best results received. $\Theta ^B$, $\Theta ^{Pri}$ and $\Theta ^{Aux}$ update in meta-learning while $\Theta ^B$ and $\Theta ^{Pri}$ are updated again in outer-loop learning. Such a design enables the meta-auxiliary learning and parameter updating assist in the primary task learning. It is equivalent to a regularization term in the primary objective learning, which also benefits the generalization in an inference with unknown data. We summarize the meta-auxiliary training algorithm in Algorithm 1.

**Algorithm 1:**
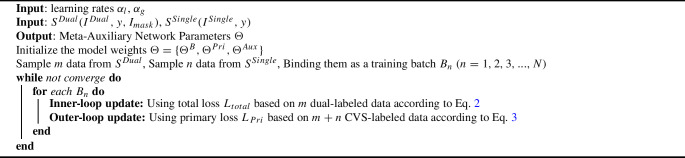
Dual-task Meta-Auxiliary Training

## Experiments and results

In this section, we introduce the experimental setting, the dataset for model development, and evaluate the multi-task results of the technique in details.

### Experimental implementation

We follow standard settings in [9, 10] to train the system in dual-task meta-auxiliary learning. For the backbone TimeSFormer, we use 6 as frame length, which is capturing 6 second surgical dynamics. The image frame is re-scaled to 224$\times$224. The batch size is set to 16. The patch is $16\times 16$ in dimension. The network is configured with 12 transformer layers, 12 attention heads, and a 768-dimensional embedding across all experiments. For auxiliary task branch, we also keep the image patch sized of $16\times 16$. Smaller numbers may achieve a more smoothed mask boundary.

For a comparison experiment, we extend the TransUnet segmentation neural network into a multi-task model by adding a multi-class recognition decoder connected with class token generated from the last transformer unit directly. Different from our proposed meta-auxiliary learning, the multi-task TransUnet simply combines CVS recognition loss and anatomy segmentation loss together. It is only trained with dual-labeled image samples using the shared CNN-transformer core.

### Dataset

Without loss of generality, we conduct the model training and evaluation on a composed robotic-assist LC dataset and a benchmark generated from tradition LC.

#### MT-CVS

To best serve clinical research and applications, we proposed a new dataset, named **MT-CVS**, that contains anatomy mask annotation and CVS labels from collected robot-assisted LCs. The dataset is curated from 106 clinical procedures covering various surgical practices worldwide using the Da Vinci ^®^ system. Every procedure video undergoes a rigorous annotation and validation process conducted by two specialists who are trained to meticulously annotate the surgical workflow. If annotation disagreement happens, a clinician supervisor will review and make the final decision on labels.

For anatomy segmentation masks, we annotate 4 major anatomies: liver, gallbladder, cystic duct and cystic artery, with 3 peripheral anatomies: the common hepatic duct, stomach and colon, for each image frame. The anatomy appearance is varied due to the process of the surgery and the viewing angle, so that not all of those classes exist for in each frame. The numbers of masks for each anatomy are unbalanced.

The CVS annotation is a multi-class binary label based on the SAGES criteria. Instead of annotating the entire surgical video which generates redundant negative CVS labels, we only annotate the video segment between the clinical steps “Dissection of calots triangle" and “Ligation of cystic duct", during which, by definition, the milestone occurs.

All image frames and labels are extracted and annotated by sampling the video recording at 1 fps and synchronized based on a universal timestamp. The images are normalized to $640\times 512$ in dimension. There are a total **4860** frames. Among that number, **965** frames have paired CVS event label and segmentation masks.

We randomly split the dataset into training, validation, and testing subsets in the proportion of 7:1:2. The images from the same procedure remain in only one set. The statistics of each category are summarized in Table 1.

**Table 1:** MT-CVS dataset statistics

**Anatomy mask**	Liver	Gallbladder	Cystic duct	Cystic artery	Common hepatic duct	Stomach	Colon
**No. of instance**	4852	4860	3803	3206	632	374	481
	**CVS label**		C1	C2	C3	CVS Any	CVS All
	**No. of instance**		2233	2248	2532	3435	1615

The entire dataset contains 4860 images. The upper two rows are statistics of annotated anatomical masks. On the same data, the bottom two rows are instance statistics of CVS label. **CVS Any** represents the number of frames containing at least one CVS criterion achieved. **CVS All** represents number of frames that containing three criteria met

#### Endoscapes

The largest public dataset satisfying the multi-task Cholecystectomy study is the Endoscapes from CAMMA [16]. To benchmark our study and provide a fair comparison, we paired Endoscapes-CVS201 with Endoscapes-Seg50 to formalize a dual-task dataset. In the dataset, there are 119,090 frames from 201 videos annotated with CVS event labels by 3 experts. We set a 0.5 threshold on the average scores to create binary CVS labels. Among the data, there are 493 frames from 50 cases with segmentation masks. We consider those frames as dual-labeled samples.

We reordered CVS criteria in the dataset to keep it consistent with SAGES definition. For the segmentation masks, we only adopt 3 anatomical classes: gallbladder, cystic artery, and cystic duct to match the same annotation ontology of our composed MT-CVS dataset. The dataset splitting follows the default according to the technique report [17]. The detailed dataset description can also be found in the report [17].

### Results and analysis

Due to the dual-task nature, the training for primary and auxiliary tasks is conducted simultaneously. At the experimental evaluation, we separate the tasks into CVS recognition and anatomy segmentation with detailed comparison and analysis.

#### CVS achievement recognition

We first evaluate our method on the primary task of CVS recognition. We report the accuracy for individual criteria predictions. In many clinical cases, confirming whether the CVS has been achieved is a key concern for surgeons, and more detailed predictions can pose a critical role. We choose precision, recall and F1 score as metrics for the CVS achievement recognition. For the baseline, we compare our model with single-task TimeSFormer framework by treating each CVS criteria as a type of action to be classified given video input. The quantitative results for two datasets are reported in Table 2, respectively. We clearly observed the encoded class token from auxiliary task indeed captured a enriched spatial context that benefiting the CVS milestone recognition by a large margin on two datasets. Our approach outperforms several state-of-the-art methods [7, 9, 10], particularly in scenarios requiring scalable data utilization.

**Table 2:** Comparison on CVS detection task with MT-TransUnet, Single Task TimeSFormer and DeepCVS [7] on MT-CVS and Endoscapes

DS	Model	C1	C2	C3	CVS Achievement
MT-CVS	MT-TransUnet	0.77	0.69	0.71	Precision: 0.67 Recall: 0.69 F1: 0.68
	TimeSFormer	0.82	**0**.**78**	0.79	Precision: 0.73 Recall: 0.77 F1: 0.75
	DeepCVS	0.74	0.61	0.66	Precision: 0.67 Recall: 0.69 F1: 0.68
	Ours	**0**.**87**	**0**.**78**	**0**.**85**	**Precision: 0.76 Recall: 0.81 F1: 0.78**
Endoscapes	**MT-TransUnet**	0.64	0.61	0.62	Precision: 0.62 Recall: 0.60 F1: 0.61
	TimeSFormer	0.70	0.65	0.69	Precision: 0.64 Recall: 0.65 F1: 0.64
	DeepCVS	0.63	0.59	0.61	Precision: 0.58 Recall: 0.61 F1: 0.60
	Ours	**0**.**73**	**0**.**74**	**0**.**77**	Precision: 0.73 Recall: 0.71 F1: 0.72

The accuracy is the metric for individual criteria achievement. Precision, recall and F1 score are reported in CVS achievement

#### Anatomy segmentation

As the auxiliary task, feature learning from the segmentation loss boosts the primary CVS recognition. At the same time, anatomy segmentation is also benefited from incorporating meta-learning via the core TimeSFormer learner. We evaluate the merit of the meta-auxiliary learning by computing the standard segmentation metrics on the auxiliary task on each generated anatomy mask. We choose the TransUnet segmentation model as the major baseline and observe that the proposed meta-auxiliary learning approach outperforms over 14.6% in average on IOU and 13.1% on Dice Score (on MT-CVS). Similar and consistent gains also achieved on Endoscapes dataset.

**Table 3:** Comparison on anatomy segmentation task with TransUnet, MT-TransUnet and DeepCVS in terms of IOU and Dice Score (at last column) metrics on MT-CVS

Model	Liver	Gallbladder	Cystic Duct	Cystic Artery	Common Hepatic Duct	Stomach	Colon	mIOU	Dice Score
TransU	0.714	0.858	0.783	0.722	0.620	0.635	0.751	0.732	0.756
MT-TransU	0.733	0.840	0.835	0.767	0.656	0.612	0.795	0.775	0.781
DeepCVS	0.685	0.680	0.623	0.603	0.554	0.589	0.593	0.624	0.692
Ours	**0**.**813**	**0**.**916**	**0**.**895**	**0**.**824**	**0**.756	**0**.**683**	**0**.**854**	**0**.**839**	**0**.**855**

**Table 4:** Comparison on anatomy segmentation task with TransUnet, MT-TransUnet and DeepCVS in terms of IOU and Dice Score (at last column) metrics on Endoscapes

Model	Gallbladder	Cystic Duct	Cystic Artery	**mIOU**	**Dice Score**
TransU	0.795	0.694	0.662	0.703	0.736
MT-TransU	0.812	0.756	0.687	0.722	0.734
DeepCVS	0.772	0.712	0.690	0.714	0.731
Ours	**0**.**858**	**0**.**790**	**0**.**725**	**0**.**778**	**0**.**822**

**Fig. 3:**
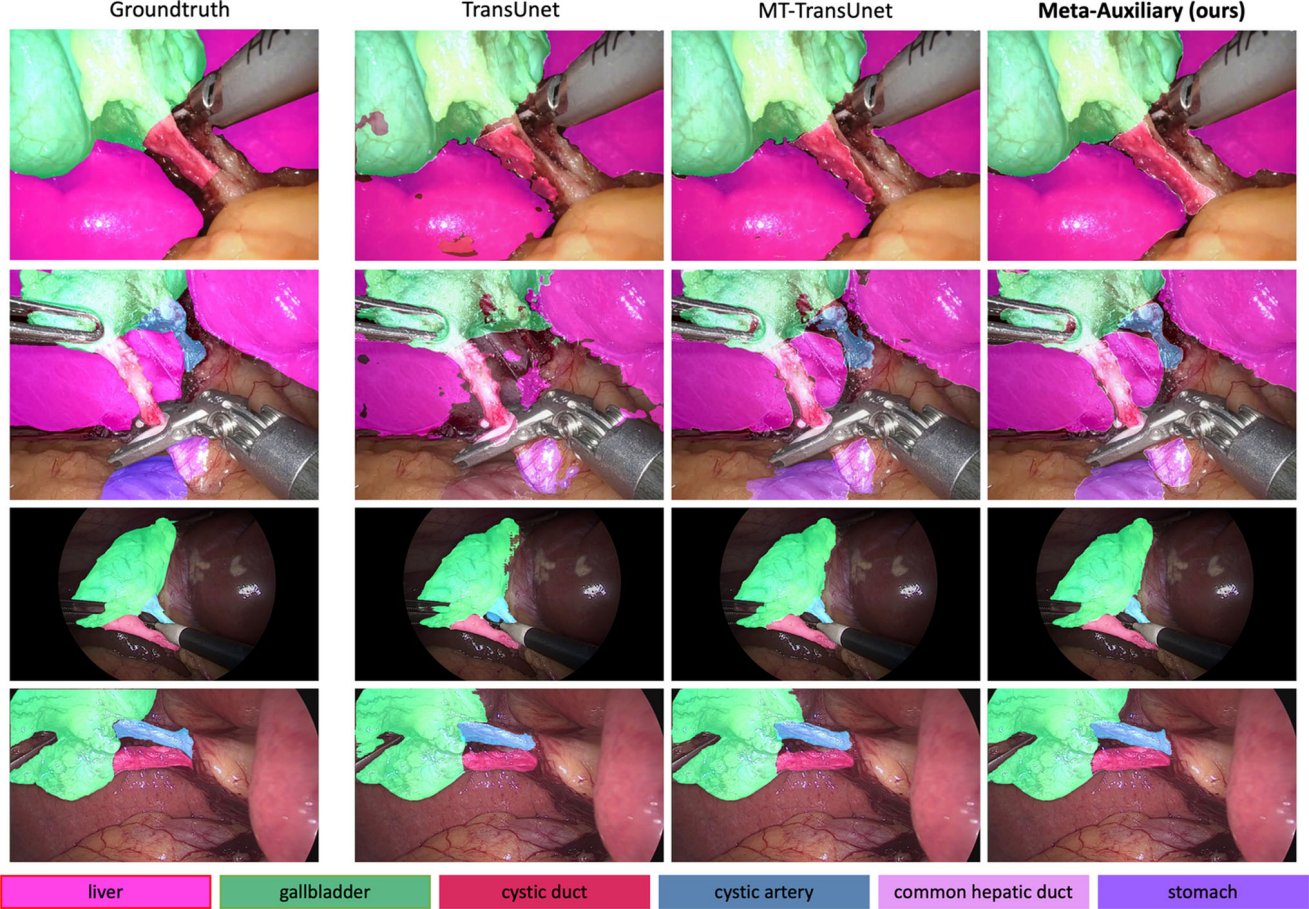
Visualized segmentation results. The top two rows are images from MT-CVS and bottom two rows are images from Endoscapes. Our proposed method (Meta-Auxiliary) is qualitatively outperformed than other state-of-the-arts

We also compare the performance with multi-task TransUnet to better understand the source of improvement by meta-auxiliary learning. We achieve more than 8% and 10% gains, respectively, on MT-CVS and Endoscapes by adopting the meta-auxiliary learning even at the auxiliary task with very limited training samples. The meta-learning via joint loss explicitly conjugates on spatial and temporal correlations in a relative long range from the input sequence, therefore, benefiting in better anatomical context understanding. The quantitative results are listed in Tables 3 and 4.

In Fig. 3, we illustrate the anatomy segmentation masks to qualitatively demonstrated the performance. All masks are the direct outputs from the auxiliary branch without post-fine-tuning.

#### Limitations

Despite the model’s proven success in the dual-task, it still struggles with challenging cases. Most failures in CVS recognition occur during the identification of Criterion 2 (C2) [7, 15]. This is primarily due to two key factors. First, C2 is inherently influenced by subjective interpretation, making consistent recognition challenging. Second, the variability in the field of view of endoscopic images further complicates the process, as it can obscure critical anatomical landmarks or alter their appearance, reducing the reliability of anatomical segmentation and CVS recognition [5, 7].

### Ablation study

The proposed meta-auxiliary learning algorithm is mainly for eliminating the negative impact of the label imbalance dataset in the multi-task challenge and boosting the primary task by harmonizing multiple learning tasks/factors with a sophisticated scheme. We observe that the proportion of the label-paired sample in a learning batch affects the model performance. In the ablation study, we varied the proportion of the label-paired samples (m) and CVS-only labeled samples (n) in a batch to testify the hypothesis on how it affects the meta-auxiliary learning. Quantitative results from experiments are summarized in Table 5.

**Table 5:** Ablation study on various proportions of the label-paired samples and CVS-only labeled samples

DataSet	Task${\backslash }$Proportion	7:1	5:3	3:5	1:7
MT-CVS	**CVS Achievement**	0.81	0.78	0.71	0.66
	**Segmentation**	0.833	0.839	0.658	0.592
Endoscapes	**CVS Achievement**	0.73	0.72	0.64	0.63
	**Segmentation**	0.781	0.778	0.612	0.523

The F1 score for CVS achievement and mIOU for segmentation are reported metrics

We observe the largest incremental performance gain achieved when the proportion changed from 3:5 to 5:3 in a learning batch. Considering the label data scarcity and the annotation cost in the clinical practice, it is much appreciated to have the proposed meta-auxiliary learning to enhance the clinical milestone recognition with limited segmentation masks.

## Conclusion and future works

We introduced an innovative meta-auxiliary learning framework to address the challenges of Critical View of Safety (CVS) detection and anatomy segmentation in Robot-Assisted Laparoscopic Cholecystectomy (RLC). This framework employs joint training on a TimeSFormer-based backbone, enabling effective knowledge sharing between primary and auxiliary tasks. By leveraging the correlated anatomy segmentation to enhance CVS detection, our approach achieves superior performance compared to existing methods. Importantly, the approach allows learning both the primary and auxiliary tasks with imbalanced label sets for the two tasks.

The framework was rigorously evaluated using the newly curated MT-CVS dataset and the benchmark Endoscapes dataset, demonstrating its effectiveness across both CVS detection and multi-instance segmentation tasks. Comprehensive qualitative and quantitative comparisons reveal that our method significantly outperforms state-of-the-art approaches. These results highlight the potential of the meta-auxiliary learning framework as a robust and scalable solution for surgical video analysis, paving the way for numerous clinical intelligence applications.

While our framework achieves significant advancements, several directions remain for further exploration. First, extending the framework to incorporate temporal modeling of entire surgical procedures could provide a richer understanding of surgical workflows and enhance milestone detection. Second, integrating unsupervised or semi-supervised learning approaches could alleviate the dependency on annotated datasets, particularly for underrepresented anatomies or rare surgical events. Third, quantifying the CVS criteria through objective measurements could reduce subjective bias in decision-making. Future models should focus on incorporating standardized metrics for CVS evaluation to ensure consistency and reliability across diverse clinical settings. These future directions aim to refine and broaden the impact of the proposed framework on surgical decision-making and procedural safety.
